# Plasmonic Modes and Fluorescence Enhancement Coupling Mechanism: A Case with a Nanostructured Grating

**DOI:** 10.3390/nano12234339

**Published:** 2022-12-06

**Authors:** Margherita Angelini, Eliana Manobianco, Paola Pellacani, Francesco Floris, Franco Marabelli

**Affiliations:** 1 Department of Physics, University of Pavia, Via Bassi 6, 27100 Pavia, Italy; 2 Plasmore S.r.l, Via Vittorio Emanuele II 4, 27100 Pavia, Italy

**Keywords:** fluorescence, surface plasmon resonance, electric field coupling mechanism, optical characterization, FDTD simulation

## Abstract

The recent development and technological improvement in dealing with plasmonic metasurfaces has triggered a series of interesting applications related to sensing challenges. Fluorescence has been one of the most studied tools within such a context. With this in mind, we used some well characterized structures supporting plasmonic resonances to study their influence on the emission efficiency of a fluorophore. An extended optical analysis and a complementary investigation through finite-difference time-domain (FDTD) simulations have been combined to understand the coupling mechanism between the excitation of plasmonic modes and the fluorescence absorption and emission processes. The results provide evidence of the spectral shape dependence of fluorescence on the plasmonic field distribution together with a further relationship connected with the enhancement of its signal. It has made evident that the spectral region characterized by the largest relative enhancement closely corresponds to the strongest signatures of the plasmonic modes, as described by both the optical measurements and the FDTD findings.

## 1. Introduction

The interest in and the importance of fluorescence measurements in sensing technology, particularly for bio-diagnostic-related problems, has been emphasized in a large part of the literature [1,2,3,4,5,6], whereby a wide variety of ways to enhance the fluorescence signal have been explored. Over the past years, the technological evolution of nanostructured materials and the possibilities of producing metasurfaces with peculiar optical properties allowed the exploitation of photonic and plasmonic systems to amplify and improve the fluorescence efficiency [7,8,9,10,11,12].

For both photonic and plasmonic structures, the enhancement is based on the electric field manipulation by using cavities [13,14,15] or surface effects, such as Bloch Surface Waves (BSW) [16,17,18,19] in the case of photonics, or the excitation of plasmonic modes, either propagating polaritons or localized, in the case of (metal-based) plasmonics [20,21,22,23].

With respect to this last scenario, several results have been reported on the exploitation of metallic nanoparticles, randomly dispersed, as well as organized into clusters or lattice structures, with different shapes and sizes [24,25,26,27,28,29,30,31,32]. Metallic nanoparticles supporting localized surface plasmon (LSP) resonances are, in general, preferred with respect to metallic layers or surfaces supporting propagating modes, called surface plasmon polaritons (SPPs), because the coupling with the excitation and emission light beam is much easier, and geometrical or momentum conservation constraints are not very important for LSP, so a large variety of configurations can be explored.

On the other hand, beyond the use of uniform layers supporting SPP modes [33,34,35], not many results have been reported for connected metallic surfaces, such as hole gratings [36,37,38]. As a matter of fact, the opto-plasmonic behavior of such kind of structures arises from the interaction among LSP and SPP modes, which exhibits a series of mixed modes, such as Fano resonances [39,40,41,42,43]. The knowledge and the control of such modes could, besides the enhancement, allow the engineering of the fluorescent emission in terms of angles and direction.

In the present work, we begin to study, both experimentally and by theoretical modelling, the fluorescence behavior of some 2D plasmonic gratings which have already shown to be a convenient platform for sensing, exploiting imaging surface plasmon resonance (SPR) detection [44]. Evidence of clear fluorescence enhancement has been obtained by measurements performed in different geometrical and optical configurations. A finite-difference time-domain (FDTD) simulation model of the plasmonic response of the studied samples has been implemented to evaluate the field distribution, in order to suggest and support an explanation of the experimental results.

The manuscript will first present the basic morphological and optical experimental characterization of the samples. Then the fluorescence results will be shown. Following the description of the FDTD model, the main simulation results will be exhibited and discussed in connection with the experimental results.

## 2. Materials and Methods

**Figure 1:**
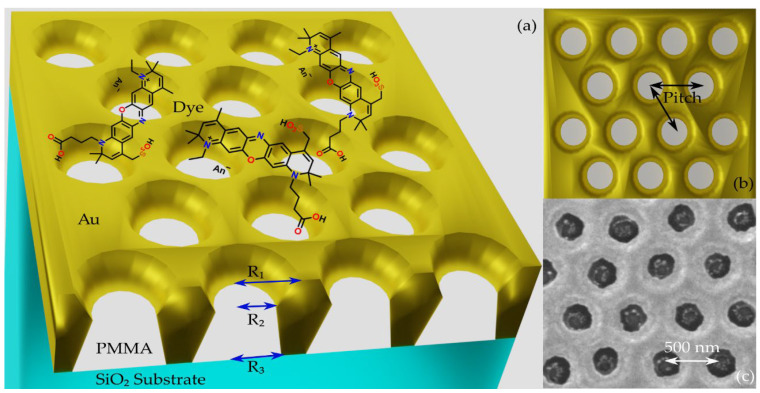
Structure of the platform under investigation: (**a**) outline of the stack showing the plasmonic metasurface, PMMA/air nanopillars in a gold layer, functionalized to deposit ATTO700 dye. The interface of the structure with the SiO_2_ substrate is referred as the back side (BS) of the structure, while the gold/PMMA/air interface as the front side (FS); (**b**) top-view of the plasmonic metasurface geometrical features determining the optical response. In our plasmonic metasurface, the presence of both LSPs and SPPs over the same spectral interval enables their mutual coupling (hybrid plasmonic resonances). Their spectral position depends on the lattice pitch because the excitation of surface plasmons is provided by the surface periodicity. Further, the array pitch is responsible for the spectral tuning of the plasmonic resonance, whereas the radii mainly influence the plasmonic resonance shape. Consequently, these parameters determine the interaction volume to maximize the spectral overlap (coupling efficiency) between the plasmonic resonance (spectra) and the dye emission (spectra); (**c**) FS SEM image of the plasmonic metasurface. The arrows indicate the dimensions of the geometrical parameters and morphological features.

The investigated plasmonic metasurface is made of hexagonally arranged poly(methyl methacrylate) (PMMA) nanopillars embedded in a gold layer on a SiO_2_ substrate (Figure 1). The nanofabrication of the plasmonic metasurface relies on a well-established protocol, based on colloidal lithography and plasma-enhanced chemical vapor deposition [43]. A detailed description of the nanofabrication process can be found in Appendix A.1. In this work, the gold/PMMA/air interface of the plasmonic metasurface (Figure 1a) will be referred to as the front side (FS), while the interface of the structure with the SiO_2_ substrate will be referred to as the back side (BS).

### 2.1. Dye Deposition

ATTO700 dye was selected to provide a distinct spectral overlap between the dye photoluminescence (PL) absorption and emission bands and plasmonic features observed in the reflectance (R) and transmittance (T) spectra of the sample under study.

The dye deposition was performed by drop-casting on the FS of the plasmonic metasurface and on a bare SiO_2_ glass slide, taken as a reference. Before the deposition process, to provide uniformity and stability, five alternating positively and negatively charged polyelectrolyte layers (PEL) were deposited on the targeted surface. The PEL coating procedure can be described as follows: the sample is immersed for two minutes in a targeted solution, then washed with MilliQ water and dried under nitrogen flow. By alternating the dipping procedure in a solution of poly(styrene) sulfonate at 2% and a solution of poly(diallyldimethylammonium) chloride at 2%, the PEL layers are stacked on the sample surface. Once a number of 5 PEL layers is reached (with a total thickness of 10 nm), a 10 μM aqueous solution of ATTO700 is deposited by drop-casting. In this way, dye molecules are supposed to form a uniform single layer over the sample surface.

### 2.2. Optical Measurements

#### 2.2.1. Variable-Angle Reflectance and Transmittance

**Figure 2:**
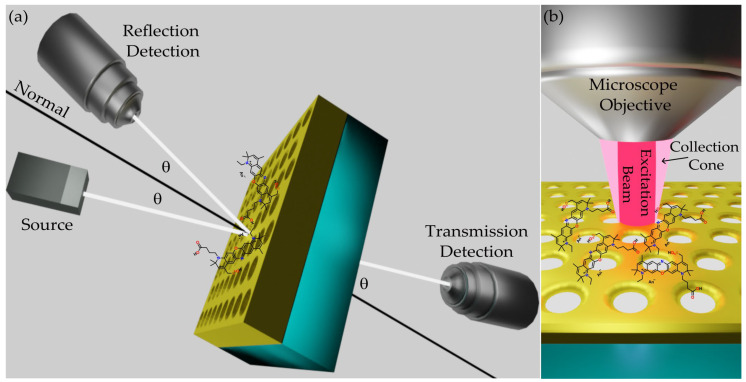
Measurement schemes. (**a**) Reflectance and transmittance outline. The R spectra were recorded in specular reflectance configuration, while the T spectra were collected by rotating the source and the detector by the same angle with respect to the normal incidence of the plasmonic metasurface. (**b**) Excitation and collection epifluorescence scheme for photoluminescence detection.

The plasmonic features of the metasurface were investigated with broadband (500 to 1100 nm) variable angle reflection and transmission measurements performed with a commercial Fourier transform spectrometer Bruker IFS66S associated with a home-made micro reflectometer in the configuration shown in Figure 2a. Owing to momentum conservation, the periodicity of the plasmonic metasurface enables the direct excitation of SPPs at the sample interface, and the related features appear in the R and T spectra. By varying the incidence angle, their energy dispersion can be measured.

The angle of incidence was varied between 2 and 62 degrees in collecting the specular reflectance spectra and between 0 and 60 degrees for the transmittance ones. Angles were varied in steps of 2 degrees, whereas the angular divergence of the light cone was less than 1 degree. A Glan Taylor polarizer was used to select TE or TM polarized light (electric or magnetic field perpendicular to the incidence plane, respectively). The characterization was performed by measuring the spectral quantities from both the FS and BS configurations.

#### 2.2.2. Photoluminescence

The PL signals were collected, as shown in Figure 2b, using a Labram Dilor spectrometer equipped with a He–Ne CW laser characterized by a 632.8 nm excitation line and nominal optical power of 15 mW. An Olympus microscope HS BX40 was used to perform measurements in epifluorescence geometry. Two objectives with 0.25 and 0.50 numerical aperture (NA) and an Optical Density 2 filter were used. The circular excitation spot was 100 µm^2^ and 5 µm^2^, respectively. A pumping power of 75 µW was measured with a power meter. Regarding the plasmonic metasurface, the PL signal was collected by focusing the excitation spot at the gold layer with both the FS and the BS facing the objective. With regard to the SiO_2_ substrate slide, the PL signal was collected by focusing the excitation spot at the dye layer also from the FS and BS. A built-in CCD Peltier-cooled camera was used to collect the PL signals in the wavelength range from 650 to 820 nm.

### 2.3. Structural Model and Simulations

An FDTD model of the plasmonic metasurface was built in Lumerical FDTD [45] to analyze and understand its optical response. The accordance between the simulated and experimental T spectra was used as a criterion to retrieve the geometrical parameters of the structural model. In the comparison with the simulation results, not only was the spectral position of the T peak observed in the experimental T spectrum at 770 nm considered, corresponding to the main LSP resonance, but also the entire spectral shape.

**Figure A1:**
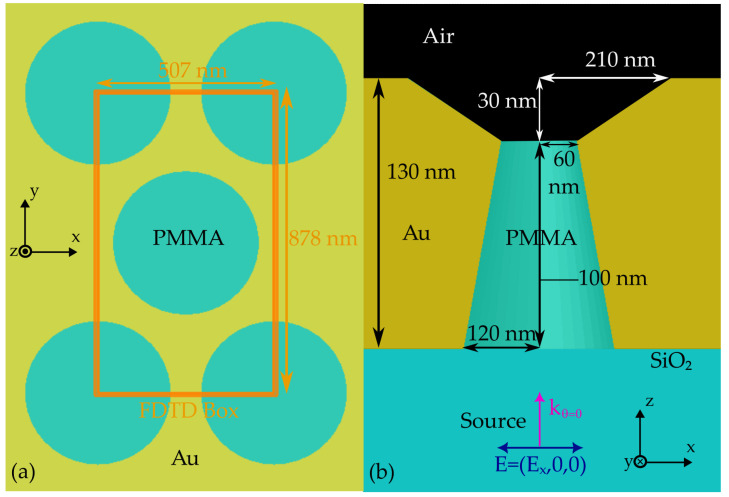
Top (**a**) and side (**b**) views of the FDTD optimized structure. The geometrical parameters of the structural model obtained from the tuning process are displayed together with their dimensions.

The metasurface was modelled in FDTD as a hexagonal lattice of PMMA nanopillars in a gold layer on a semi-infinite SiO_2_ substrate. In a first process, the array pitch and gold thickness were fixed according to nanofabrication parameters at 500 nm and 120 nm, respectively. The nanopillars shape was modelled considering the compatibility with the nanofabrication process and SEM characterization (Figure 1c). The FDTD nanopillar structure was built as a truncated cone of air stacked onto a PMMA nanopillar, according to Figure A1 in the Appendix. The radii of the air truncated cone (R1, R2, Figure 1a) were initially fixed from the values of the inner and outer radii of the hole structure measured with SEM at the FS of the metasurface (Figure 1c). A customized sweep script was implemented in Lumerical FDTD [45] to recover the PMMA nanopillar radius at the BS (R3, Figure 1a). By varying R3, the resulting transmittance spectrum was compared with the experimentally obtained spectrum until a good tuning of the peak at 770 nm was reached. After that, the previously fixed geometrical parameters were slightly varied to obtain a finer tuning of the T spectral shape. The simulated T spectrum has a well-tuned peak at 770 nm, and a slightly narrower spectral shape with respect to the experimental spectrum, while the broader structure at shorter wavelengths appears red-shifted and with lower intensity. This discrepancy is acceptable resorting to the tuning criterion adopted for the simulated T spectrum, considering some structural disorder affecting the experimental results. The agreement between the T spectra of the optimized structure and the experimental spectra, both in terms of spectral shape and feature position, as well as the compatibility of the geometrical parameters with the SEM reference values, support the reliability of the model adopted to mimic the plasmonic metasurface and provide the possibility to compute the electric fields. The geometrical parameters resulting from the mutual analysis of SEM images, together with the simulation outcomes, are R1 = 210 nm, R2 = 60 nm, R3 = 120 nm, a gold thickness of 130 nm and pitch of 507 nm.

In this way, a deeper insight into the plasmonic response can be achieved when studying the electric field (EF) distributions and spectral profiles, calculated from the optimized FDTD structure. Various 2D power monitors were used to compute the electric field expansion in both real space and wavelength at selected crucial planar cross-sections.

An additional script was implemented to extract the EF magnitude (M), defined as

(1) M(EF(x,y,z,λ))=Re(|Ex(x,y,z,λ)|2+|Ey(x,y,z,λ)|2+|Ez(x,y,z,λ)|2), 

for x-normal and y-normal planes centered in the origin of the FDTD box coordinate system, and a z-normal monitor placed in correspondence with the air/PMMA/gold line of contact. By integrating Equation (1) in space, the EF magnitude spectral profile at the gold/PMMA/air interface was calculated. The EF spectral profile and the experimental T spectrum, displaying both the narrower structure centered at 770 nm and the broader one at shorter wavelengths, provide consistent validation of the structural model and give a sufficiently accurate description of the real system for the purpose of the present study.

## 3. Results

**Figure 3:**
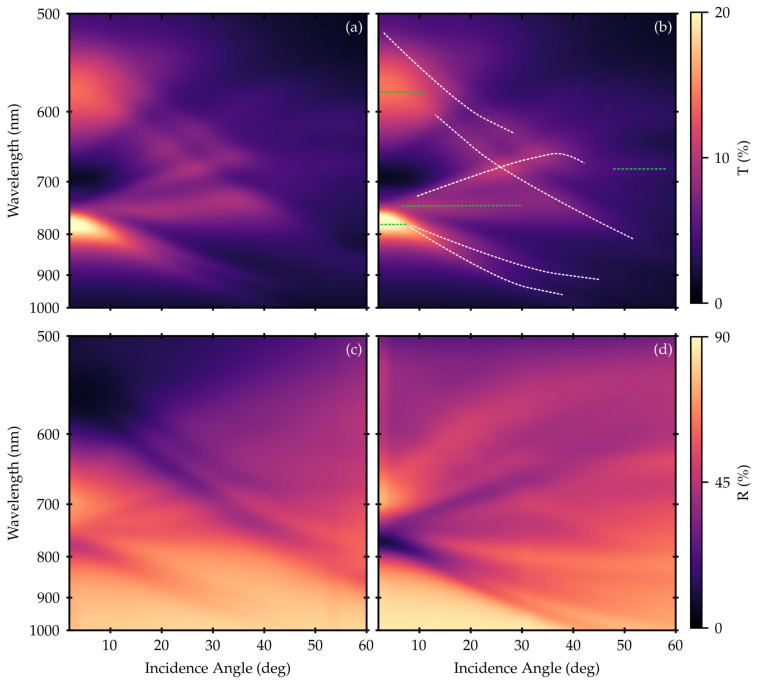
Bidimensional map of T (**a**,**b**) and R (**c**,**d**) of the plasmonic metasurface with respect to the incidence angle. In panels (**a**,**c**), the source illuminated the structure from the front side (FS), while in panels (**b**,**d**), this occurred from the back side (BS). In panel (**c**) the dashed lines provide an indication of the more localized (green) and more dispersive (white) nature of the complex hybrid modes resulting from the mixing of LSPs and SPPs occurring in this metasurface. A comprehensive study about the hybrid modes dispersion in such metasurfaces can be found in [43].

Reflectance and transmittance data are plotted in the form of an intensity map as a function of both the incidence angle and the wavelength in Figure 3.

It is evident that the optical features of both R and T spectra show strong dispersion effects. In addition, a correlation between R maxima and T minima, and vice versa, is noticeable. The spectral features follow the dispersion of the SPP modes at the gold/air and gold/SiO_2_ interfaces, respectively, compared to calculated ones. They provide evidence of the coupling of the electromagnetic (EM) wave component projected onto the surface with the polariton modes. The observed behavior has already been described in previous work dealing with the excitation of plasmonic modes occurring in this kind of surface [41,42,43].

The T data are almost independent of the incidence direction. On the other hand, from the air side (FS) towards the glass substrate (BS), or vice versa, R spectra exhibit a different sensibility of the dispersion features related to the gold/air or the gold/SiO_2_ interface.

It is worth noting that the strongest observed feature, related to the interplay of SPP and LSP modes, occurs between 700 and 800 nm at the lowest angles of incidence.

**Figure 4:**
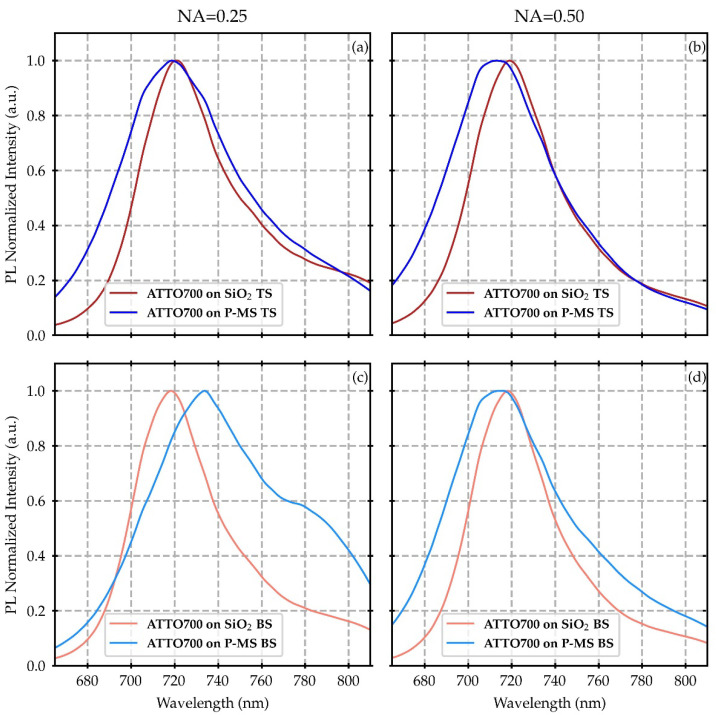
Normalized photoluminescence spectra of ATTO700 dye deposited on the top side of the plasmonic metasurface (P-MS) and the reference SiO_2_ slide. The first row refers to the excitation and collection from the FS for microscope objective numerical apertures of NA = 0.25 (**a**) and NA = 0.50 (**b**). Panels (**c**,**d**) refer to the excitation and collection from the BS for microscope objective numerical apertures of NA = 0.25 and NA = 0.50, respectively.

The normalized PL spectra of ATTO700, collected from the plasmonic metasurface and the glass reference, are shown in Figure 4 for NA = 0.25 (corresponding to a maximum solid angle of excitation/collection of 15°), NA = 0.50 (corresponding to a maximum solid angle of excitation/collection of 30°), and for both epifluorescence excitation conditions (FS and BS), as reported in the Materials and Methods section. The two chosen NA values were selected (i) to efficiently focalize the excitation spot on both the upper and lower interfaces of the samples, thanks to a proper focal depth; (ii) to collect the PL emission, taking into proper account its angular dependence due to the interaction of the dye excitation/emission with the plasmonic modes, characterized by a strong angular dispersion (Figure 3). Then, the selection of a low NA enables us to focus on the contribution of the lower angle features, together with the large resonances at 650 nm and 780 nm. On the other hand, a higher NA value enables the collection of the contribution of a larger number of plasmonic modes, mainly related to the plasmonic band gap opening, but wider distributed both in the spectrum and in angles.

The normalized emission spectra of the SiO_2_ reference samples do not show a dependence either on the optical configuration or on the microscope objective NA. Both spectral shape and wavelength tightly correspond to the literature data of the ATTO700 dye (as reported in Appendix A.3).

Instead, a different behavior is observed for the dye emission deposited on the plasmonic metasurface. In this case, in general, a spectral shift and a remarkable broadening of the line shape can be easily observed with respect to the measurements on the glass reference.

The spectra measured from the FS configuration mainly increase their spectral weight at the lowest wavelengths.

Regarding the BS configuration and with a high NA, the line-shape broadening is more symmetrical with the relative intensity increasing both at the lower and larger wavelengths, with respect to the maximum.

In contrast, for the BS configuration and with a low NA, a large enhancement can be observed for the largest wavelengths, with a shoulder emerging at about 780 nm, closely corresponding to the maximum in T spectrum (at low incidence angles).

**Figure A2:**
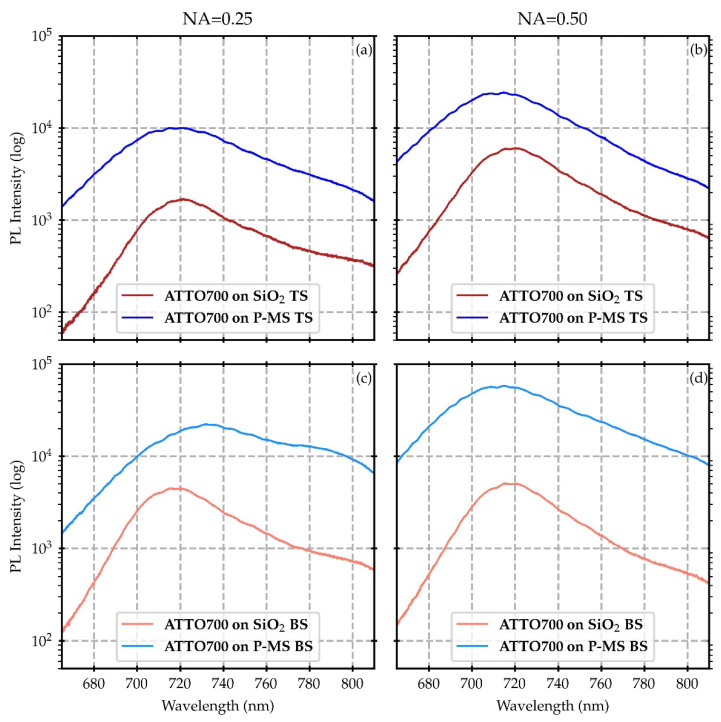
Photoluminescence spectra of ATTO700 dye deposited on the top side of the plasmonic metasurface (P-MS) and on the SiO_2_ reference slide. First row refers to the excitation and collection from the FS for microscope objective numerical apertures of NA = 0.25 (**a**) and NA = 0.50 (**b**). Panels (**c**,**d**) refer to the excitation and collection from the BS for microscope objective numerical apertures of NA = 0.25 and NA = 0.50, respectively.

Moreover, this last configuration exhibits the largest enhancement, even in terms of absolute intensity (Figure A2).

## 4. Discussion

Considering both the ATTO700 dye absorption and emission PL features together with the analysis of the R and T behavior contained in the maps plotted in Figure 3, we identified four interesting wavelength points useful to characterize the role of the EF in defining the optical response of the plasmonic metasurface, and its interplay with the fluorophore.

These four points are the laser excitation wavelength used to pump the ATTO700 dye set at 632 nm, the nominal peak in the ATTO700 dye PL emission signal located at 720 nm, the minimum in the T spectra indicating the opening of the plasmonic bandgap placed at 700 nm and the peak related to the main LSP mode tuned at 770 nm.

**Figure 5:**
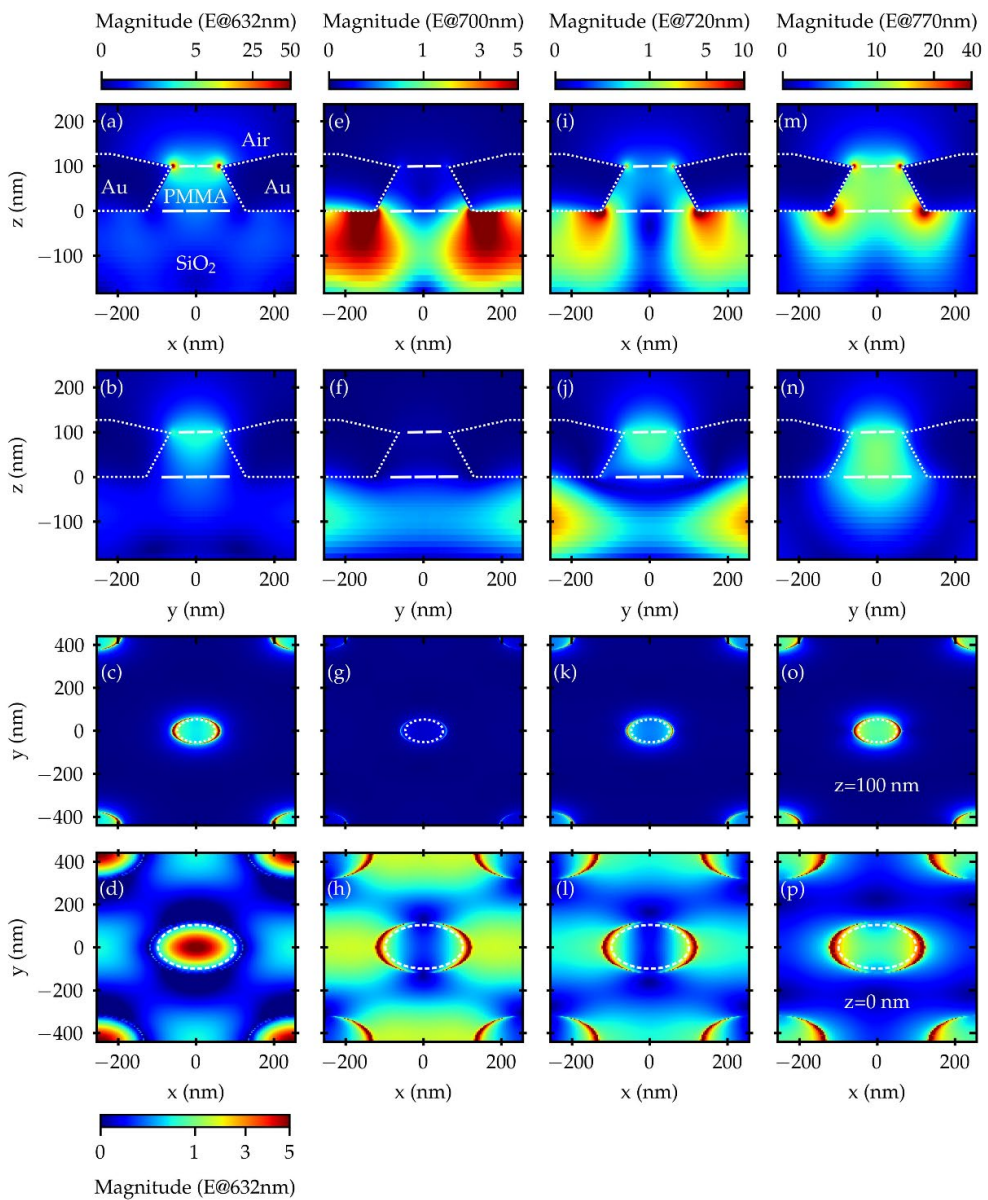
Electric field expansion in the PMMA nanopillar proximity region calculated with Ansys FDTD Lumerical software for different wavelengths: 632 nm (panels (**a**–**d**)), 700 nm (panels (**e**–**h**)), 720 nm (panels (**i**–**l**) and 770 nm (panels (**m**–**p**)). Panels (**c**,**g**,**k**,**o**) and panels (**d**,**h**,**l**,**p**) refer to the EF expansion calculated at the nanopillar top (z = 100) and bottom (z = 0 nm), respectively. It is worth emphasizing the change in the magnitude scale corresponding to panel (**d**) with respect to panel (**c**).

Resorting to 3D-FDTD simulations (see Figure 5), we calculated the EF expansion at these four wavelengths in the PMMA nanopillar proximity region considering an x–z and y–z vertical cross-section to capture the interaction between the plasmonic metasurface and the plane wave light source, identifying the position of plasmonic hot spots. The x–y horizontal cross-sections were also evaluated, clearly depicting the EF role and interplay effect in the definition of the T, R and PL signals.

Examining the EF expansion at 632 nm in panels (a) to (d) in Figure 5, the structure can, in principle, support the laser pumping process via a circular hot spot placed at the nanopillar top (at z = 100 nm in the simulation model), possibly increasing its efficiency. In any case, the excitation of the dye at the pumping wavelength is well supported by the plasmonic field, either when the light is impinging from the FS or the BS of the metasurface. In addition, the ability of the PMMA truncated cone to act as a tapering structure can be considered. This is indicated by the absence of a hot spot at the nanopillar bottom, in conjunction with the remarkable value of the maximum intensity for the magnitude of the EF, whose effect can be seen in the increase in the T signal at wavelengths below the bandgap, as shown in panels (a) and (b) in Figure 3. This taper shape then creates a type of optimized directional channel which is able to simultaneously boost the transmission of the EF from the back side of the structure towards the front side where the dye molecules have been deposited, and squeeze the EF in the right portion of the structure compatibly with the promotion of their absorption efficiency.

**Figure 6:**
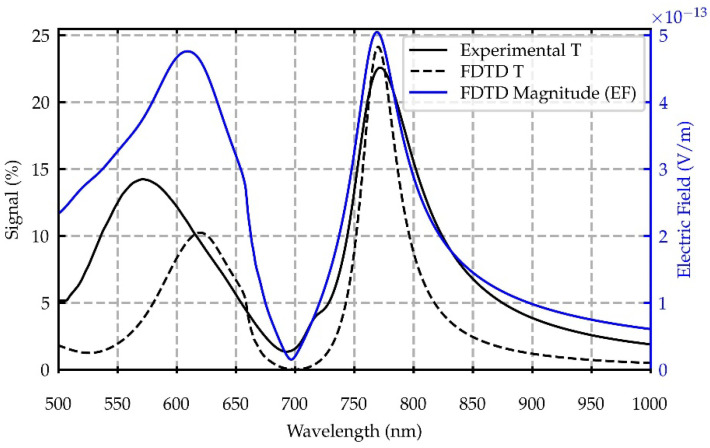
Comparison between experimental and simulated transmittance spectra at normal incidence, computed in the x–y horizontal plane placed at z = 530 nm together with the electric field magnitude evaluated in the x–y horizontal plane placed at z = 130 nm. Both z coordinates correspond to the gold/SiO_2_ interface.

From the analysis of the EF expansion at 700 nm in panels (e) to (h) in Figure 5, it can be inferred that the plasmonic structure primarily acts as a mirror. This statement is also confirmed by the minima in the T spectra and maxima in the R spectra reported in Figure 3. The incident EF interacts with the structure mainly at the SiO_2_ substrate/gold interface, but only a small fraction of it is transmitted from the BS to the FS. As a matter of fact, this statement is also consistent with the corresponding values reported for the EF magnitude typical of the reflection process, with a remarkable field value just on the BS (recall that the simulation was performed with the plane wave source impinging from the BS). As a result, the wavelength filtering effect deriving from the metasurface plasmonic-driven diffraction capabilities can be even more appreciated looking at the comparison between the experimental and simulated T spectra at normal incidence, as depicted in Figure 6. The integrated EF magnitude, at the top gold/air interface of the plasmonic metasurface, was plotted as a function of wavelength. This plot gives an indication of (i) the level of agreement between the measured and simulated T curves, and (ii) their behavior in following the EF spectral evolution.

The other remarkable wavelength point is represented by the main LSP resonance tuned at 770 nm. As shown in Figure 3, this point, in any case lying within the PL emission range of the fluorophore, is characterized by the maximum intensity in T. In this case, the EF expansion (Figure 5, panels (m) to (p)) assumes the shape of a two-paired circular hot spot placed at the nanopillar top (at z = 100 nm in the simulation model) and at the nanopillar bottom (at z = 0 nm in the simulation model), possibly favoring the EF and energy exchange between the two sides of the structure. As a result, it is reasonable to hypothesize that the PL emission of the ATTO700 dye might be collected by the upper circular hot spot and resonantly conveyed into the lower circular hot spot, to be selectively emitted more efficiently towards the BS of the structure. This is also consistent with the considerations of Geddes and Lakowicz in [34].

Finally, the last wavelength point coincides, for completeness, with the ATTO700 dye PL emission peak at 720 nm. In this case, from the R and T maps in Figure 3, the plasmonic structure is in a transitional region, from the bandgap at 700 nm to the main LSP resonance at 770 nm, with no other resonances in this range. This behavior is reflected in the corresponding EF expansion (Figure 5, panels (i) to (l)), which preserves the shape of a pair of circular hot spots. Nevertheless, since we are slightly off the LSP resonance, the hot spot at the FS appears with the same symmetry, but with a much lower magnitude. On the other hand, the less localized nature of the hot spot at the BS, which appears expanded in the substrate region, reflects the vicinity of 720 nm to the spectral filtering region at the bandgap centered at 700 nm.

For completeness, it is useful to underline additional interesting information that can be evaluated by a deep analysis of the curves compared in Figure 6.

The first feature is the conformity in the shape between the measured and the simulated T curves. This provides positive feedback about the reliability of our customized optimization script and consequent FDTD model built for the simulation activity, sustaining, in addition, the reliability of the EF expansions. Therefore, it is interesting to consider the conformal behavior of the simulated T with respect to the simulated magnitude of the EF at the nanopillar top. As a result, it can be inferred that the measured T is able to provide a preliminary assessment of the EF spectral distribution. Moreover, the nanopillar top is exactly the place where the dye layer was deposited. Thus, the field distribution has a direct impact on the fluorescence process.

Driven by these considerations, it is possible to analyze the spectral behavior of the measured PL spectra of ATTO700 on the plasmonic metasurface in terms of plasmonically driven EF effects by a direct comparison with the experimental T spectra. To do so, we considered the ratio of the normalized PL spectrum of the dye on the plasmonic metasurface and on the SiO_2_ reference. Then, we compared such a ratio with the average of the T spectra performed over the angles lying within the microscope objective NAs.

**Figure 7:**
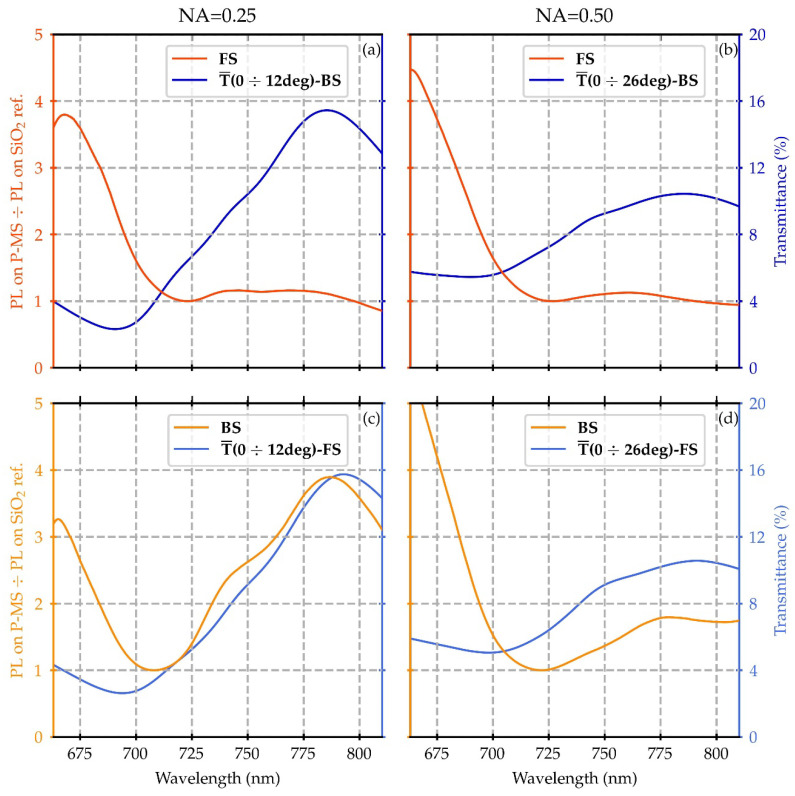
Comparison between the ratio of the normalized photoluminescence spectra of ATTO700 measured on the plasmonic metasurface (P-MS) and on the SiO_2_ reference with the transmittance signals averaged over the angles lying within the NA value. The first row refers to the positioning of the detector at the front side (FS) for NA = 0.25 (**a**) and NA = 0.50 (**b**). In the second row, the positioning of the detector is at the back side (BS) of the structure for NA = 0.25 (**c**) and NA = 0.50 (**d**).

The results of the analysis are summarized in Figure 7.

Specifically, when considering NA = 0.25 the T signal is well structured showing a minimum around 700 nm and a maximum around 780 nm, consistent with the corresponding values shown in Figure 3. On the other hand, for NA = 0.50, the T signal appears broadened, as the average of the spectra is performed over a wider range of angles. This effect can be also ascribed to an increasing spectral contribution of the coupling of the metasurface plasmonic modes, together with the presence of a growing number of EF components.

Focusing on the PL ratio curves, the data, collected as described in the Material and Methods sections, have been categorized depending on the sample side.

The FS configuration is characterized by a PL ratio bigger than unity in the region below the fluorophore PL emission peak at 720 nm, indicating an enhancement of the ATTO700 dye PL signal when deposited on the plasmonic structure with respect to the fluorophore deposited directly on the SiO_2_ reference. On the other hand, only a small variation can be observed in the wavelength region above the bandgap at 700 nm.

The BS configuration shows similar behavior with respect to the FS configuration in the region of 700–720 nm (bandgap and PL emission peak). On the contrary, the behavior is radically different in the region above this wavelength. In this case, in the PL ratios there provide evidence of a second region of enhancement of the PL signal when deposited on the plasmonic metasurface with respect to the dye deposited directly on the SiO_2_ reference. Different from the FS configuration, in the BS configuration, the ratio’s spectral behavior is conformal and reproduces the shape of the T spectra. Instead, the transition region (700–720 nm) exactly corresponds with the low field (filtering) region identified by the T minima and as shown in Figure 7, it is also close to the PL ratios’ minima. It is worth highlighting that the dye PL absorption peak is located at 700 nm. Hence, the dye molecules are being shielded from a resonant pumping stimulus, which gives us the possibility to highlight the selection in the EF transmission operated by the plasmonic metasurface.

Moreover, the described performances are consistent with the EF expansions plotted in Figure 5 and described previously. The modes that are most efficiently supported by the structure on its surface are located in the region below the band gap at 700 nm, providing an optimal configuration for coupling with fluorophore pumping. The EFs corresponding to the modes localized in the region with a wavelength greater than 700 nm nevertheless show a non-negligible component localized to the surface, but are concentrated more at the SiO_2_ substrate/PMMA/gold interface, conveying the fluorophore emission, and favoring its directionality from the side of the substrate.

## 5. Conclusions

By merging reflectance, transmittance and photoluminescence measurements, in combination with 3D-FDTD simulations, we have identified and investigated the field coupling mechanism behind the spectral shape dependence of the ATTO700 dye fluorescence emission on the plasmonic field distribution of a metastructured surface, together with its relationship with the enhancement of the signal.

In particular, by analyzing the transmittance behavior with respect to the electric field features, we have found that the spectral region characterized by the largest relative enhancement closely corresponds to the strongest signatures of the plasmonic modes.

In addition, the field coupling mechanism is, interestingly, characterized by a specific directionality for the electric field propagation, from the substrate backside towards the topside nanostructured surface and vice versa, enabling an efficient engineering of the structure in terms of pumping and collection suitable for sensing applications.

This is particularly useful when considering a practical use for our metasurface in the development of a sensing device based on surface enhanced fluorescence. In this framework, the compatibility of the measurement and functionalization schemes with microfluidic setups is crucial. Our structure is able to undertake pumping and collection from the substrate backside, which keeps the nanostructured surface completely available for functionalization and interfacing with the liquid to be analyzed, making our metasurface fully compatible with the aforementioned microfluidic requirements.

In conclusion, we consider this study to be very promising for further development aiming to realize concrete applications within the disruptive technology scenarios.

## Data Availability

Not applicable.

## Appendix A

### Appendix A.1. Nanofabrication Process

The nanofabrication process can be summarized as follows: poly(methyl methacrylate) (PMMA) dissolved in anisole is deposited by spin coating on a commercial glass substrate, forming a layer of controlled thickness. After an annealing process at 180°, to remove residual solvent and harden the PMMA layer, commercial polystyrene monodisperse spherical beads with a 500 nm nominal diameter are deposited on the water surface of a rectangular Langmuir–Blodgett trough. In this way, the spherical beads arrange in hexagonal close-packed arrays forming a 2D crystal with fixed lattice spacing. Then, the formed arrangement is transferred onto the PMMA layer by dip coating, with the PMMA layer acting as a sacrificial mask to reactive O_2_ plasma etching exposition. By controlling the etching parameters, a final tuning of the beads’ diameter is possible, creating the pattern with the desired structural features.

The obtained sample is coated by a thin gold film with physical vapor deposition to fill the etched walls in the PMMA layer.

After the polystyrene beads have been lifted off the metal-coated sample in an ultrasonic bath, the substrate is annealed at high temperature. The resulting structure is a gold film perforated by truncated cone-shaped polymeric nanopillars (Figure 1) arranged in a hexagonal array.

### Appendix A.2. FDTD Parameters

The optical properties of the gold layer are defined through the built-in Johnson and Christy material database of Lumerical [45], while the PMMA and SiO_2_ were set as perfect dielectric materials with a refractive index of 1.48 and 1.50, respectively. A linearly polarized plane wave source at normal incidence with electric field oscillations along the x-direction and a spectral interval spanning from 500 to 1100 nm is used to illuminate the structure from the bottom side (BS). Two power monitors, one placed behind the light source and one located above the FS characterized by the same spectral interval and frequency points with respect to the source, were used to collect the R and T signals, respectively. After performing convergence tests, the mesh type was set to auto non-uniform at a mesh accuracy of level 5, with an additional override mesh of 1 nm along the x-, y- and z-direction. Mesh refinement was chosen as conformal variant 2. The FDTD box boundary conditions were set to anti-symmetric along x, symmetric along y and perfectly matched layers (PML) along z. A Selecta Z690 desktop equipped with a liquid-cooled Intel^®^ Core 12th generation I7-12700K (12 core) and 64 GB of RAM was used to perform the FDTD simulations. An average simulation time of about 8 h was required to complete a single simulation.

### Appendix A.3. Fluorophore Specifics and PL Spectra

ATTO700 dye is produced by ATTO-TEC GmbH. ATTO 700 is a zwitterionic dye with a net electrical charge of zero. The relative absorption and emission spectra and further information can be found in [46].

Figure A2 shows the measured PL spectra of ATTO700 dye deposited on the FS plasmonic metasurface and those of the dye deposited on the SiO_2_ reference sample for the different epifluorescence conditions, namely impinging with the pump exciting from the FS and from the BS of the samples.
